# Применение тоцилизумаба при тяжелой эндокринной офтальмопатии с оптической нейропатией, рефрактерной к глюкокортикоидам, в клинической практике

**DOI:** 10.14341/probl13580

**Published:** 2026-01-18

**Authors:** Н. Ю. Свириденко, Е. В. Ананичева, В. М. Гершевич, Я. О. Груша, К. А. Чепилев, А. В. Суров, В. В. Юргель, К. С. Щукин, Е. Г. Бессмертная, О. А. Билевич

**Affiliations:** Национальный медицинский исследовательский центр эндокринологии им. академика И.И. ДедоваРоссия; Endocrinology Research CentreRussian Federation; Городская клиническая больница №1 им. А.Н. Кабанова; Клиническая офтальмологическая больница им. В.П. ВыходцеваРоссия; City Clinical Hospital No. 1 Named After A.N. Kabanov; Clinical Ophthalmologic Hospital named after V.P. VykhodtsevRussian Federation; Омский государственный медицинский университетРоссия; Omsk State Medical UniversityRussian Federation; Научно-исследовательский институт глазных болезней им. М.М. КрасноваРоссия; Research Institute of Eye Diseases Named After M.M. KrasnovRussian Federation; Клиническая офтальмологическая больница им. В.П. ВыходцеваРоссия; Clinical Ophthalmologic Hospital named after V.P. VykhodtsevRussian Federation; Омский государственный медицинский университет; Клиническая офтальмологическая больница им. В.П. ВыходцеваРоссия; Omsk State Medical University; Clinical Ophthalmologic Hospital named after V.P. VykhodtsevRussian Federation; Городская клиническая больница №1 им. А.Н. КабановаРоссия; City Clinical Hospital No. 1 Named After A.N. KabanovRussian Federation; Городская клиническая больница №1 им. А.Н. Кабанова; Омский государственный медицинский университетРоссия; City Clinical Hospital No. 1 Named After A.N. Kabanov; Omsk State Medical UniversityRussian Federation

**Keywords:** эндокринная офтальмопатия, оптическая нейропатия, метилпреднизолон, рефрактерность к глюкокортикоидам, тоцилизумаб, Graves’ orbitopathy, thyroid eye disease, optic neuropathy, methylprednisolone, glucocorticoid resistance, tocilizumab

## Abstract

Эндокринная офтальмопатия (ЭОП) — аутоиммунная патология тканей орбиты, ассоциированная с аутоиммунной патологией щитовидной железы. Наиболее часто она сочетается с болезнью Грейвса. Клиническая картина ЭОП чрезвычайно разнообразна, варьирует от легкого поражения орбиты до потенциально угрожающей зрению оптической нейропатии. Ранняя диагностика активной фазы ЭОП имеет важное значение, поскольку иммуносупрессивная терапия оказывает свое действие только в активной фазе, в то время как лечение пациентов в неактивной фазе включает только реабилитационную хирургию. Высокодозные внутривенные глюкокортикоиды являются первой линией лечения пациентов с умеренной и тяжелой активной ЭОП. Глюкокортикоиды широко используются и обладают противовоспалительной и иммуносупрессивной активностью, но около 20–30% пациентов остаются резистентными к введению глюкокортикоидов. Одним из перспективных направлений в лечении ЭОП, резистентной к глюкокортикоидам, являются моноклональные антитела, направленные против определенных эпитопов антигена. Нами представлен клинический случай применения тоцилизумаба при тяжелой ЭОП с оптической нейропатией, рефрактерной к глюкокортикоидам.

## Abstract (en)

Thyroid eye disease (TED) is an autoimmune pathology of the orbital tissues associated with autoimmune thyroid disease, most commonly occurring with Graves’ disease. The clinical presentation of TED is extremely diverse, ranging from mild orbital involvement to potentially vision-threatening optical neuropathy. Early diagnosis of the active phase of TED is crucial, as immunosuppressive therapy is effective only during this phase, while treatment for patients in the inactive phase consists only of rehabilitative surgery. High-dose intravenous glucocorticoids are the first-line treatment for patients with moderate to severe active TED. Glucocorticoids are widely used due to their anti-inflammatory and immunosuppressive properties, but about 20–30% of patients remain resistant to glucocorticoid treatment. One promising direction in the treatment of glucocorticoid-resistant TED is the use of monoclonal antibodies targeting specific antigen epitopes. We present a clinical case of tocilizumab treatment in severe glucocorticoid-resistant TED complicated by optic neuropathy.

## АКТУАЛЬНОСТЬ

Эндокринная офтальмопатия (ЭОП; орбитопатия Грейвса) — аутоиммунное заболевание, поражающее орбитальные ткани: ретробульбарную клетчатку (РБК) и глазодвигательные мышцы (ГДМ) [1][2]. ЭОП возникает примерно у 25% пациентов с болезнью Грейвса (БГ) и является самым частым сопутствующим ей экстратиреоидным заболеванием [1–4]. Значительно реже она развивается при аутоиммунном тиреоидите (АИТ) и эутиреозе.

В основе патогенеза ЭОП лежат перекрестные иммунные реакции между антигенами щитовидной железы (ЩЖ) и орбитальных тканей, обусловленные распознаванием рецептора тиреотропного гормона (рТТГ) и рецептора инсулиноподобного фактора роста 1 (рИФР 1) в качестве аутоантигенов на поверхности фибробластов. В результате активации рецепторного комплекса происходит дифференцировка фибробластов в миофибробласты или адипоциты, что приводит к увеличению в объеме мягких тканей орбиты [5][6]. Воспалительные реакции, возникающие при ЭОП, являются результатом сложного взаимодействия между клеточными и гуморальными иммунными механизмами. Активация Т- и В-лимфоцитов приводит к нарушению баланса цитокинов, что усиливает секрецию гиалуроновой кислоты, мукополисахаридов, гликозаминогликанов и вызывает отек РБК и ГДМ. В результате сдавления зрительного нерва (ЗН) и нарушения кровообращения развивается оптическая нейропатия (ОН) — осложнение, угрожающее зрению, которое возникает примерно у 3–8% пациентов с ЭОП [2][7][8]. Диагностируется ОН на основании клинических, офтальмологических и инструментальных исследований. Снижение остроты зрения, нарушение цветовосприятия, дефекты поля зрения, отек/бледность диска ЗН и афферентный зрачковый дефект указывают на ОН [2][7][9]. Магнитно-резонансная томография (МРТ) и/или компьютерная томография (КТ) используются для оценки компрессии ЗН увеличенными ГДМ (апикальный синдром) или для визуализации натяжения ЗН [2][7][9][10].

Согласно рекомендациям Европейской группы по изучению орбитопатии Грейвса (EUGOGO) в 2021 г., а также Американской (ATA) и Европейской (ETA) тиреоидных ассоциаций в 2023 г., первой линией терапии ЭОП, осложненной ОН, является проведение пульс-терапии высокими дозами метилпреднизолона (МП). В случае отсутствия эффекта в течение 1–2 недель или прогрессирования клиники ОН рекомендуется срочная декомпрессия орбиты [11][12][13][14]. Отсутствие реакции на системные глюкокортикоиды (ГК) (20–30%), рецидив после отмены терапии ГК (10–20%), противопоказания или нежелательные реакции во время лечения, клиническое ухудшение на фоне терапии первой линии требуют применения терапии второй линии [6][11][12]. Однако при ОН альтернативные стратегии лечения отсутствуют, и лишь ограниченное число исследований было посвящено терапии ОН, в основном ретроспективных, с небольшим размером выборки. Так как оптимальная терапия должна применяться незамедлительно из-за риска атрофии ЗН, лечение пациентов с ОН остается серьезной проблемой.

При рефрактерной ЭОП с переменным успехом применяются разные подходы к лечению. В последние годы растет интерес к таргетной терапии моноклональными антителами. Одним из перспективных препаратов для пациентов с ЭОП, резистентных к ГК, является моноклональное рекомбинантное гуманизированное антитело к рецепторам интерлейкина-6 (ИЛ-6) тоцилизумаб (ТЦЗ). ИЛ-6 — провоспалительный цитокин, концентрация которого повышается у пациентов в активной фазе ЭОП. Благодаря активации Т- и В-клеток ИЛ-6 стимулирует адипогенез и синтез гликозаминогликанов, способствуя увеличению объема тканей орбиты [1–4][8][9][15]. ТЦЗ, согласно рекомендациям EUGOGO 2021 г. и ATA/ETA 2023 г., может рассматриваться как препарат второй линии для лечения активной ЭОП средней и тяжелой степени [11][12].

Опубликовано семь отчетов о 14 пациентах с ЭОП и ОН (16 орбит), которые получали терапию ТЦЗ [8][16–20]. Также недавно представлены результаты проспективного когортного исследования среди 13 пациентов с ЭОП и ОН с компрессией ЗН, где применение ТЦЗ оказалось весьма эффективным [21]. Наш клинический случай описывает успешное применение ТЦЗ у пациентки с тяжелой ЭОП и ОН, резистентной к ГК.

## ОПИСАНИЕ СЛУЧАЯ

Пациентка К., 41 г., в апреле 2023 г. обратилась к офтальмологу «Клинической офтальмологической больницы им. В.П. Выходцева» с жалобами на дискомфорт в глазах, выраженный отек век, постоянное двоение, ограничение подвижности глаз, слезотечение, нечеткость, снижение остроты зрения, утомляемость. Из анамнеза: осенью 2022 г. похудела на 10 кг за 4 месяца при хорошем аппетите, появились эпизоды учащенного сердцебиения, утомляемость. С декабря 2022 г. — отек век, спонтанные боли в глазах эпизодически; с января 2023 г. — ограничение подвижности глаз, двоение. Обратилась к офтальмологу поликлиники, диагностирована ЭОП, детально не обследована. Острота зрения (далее везде — максимально корригированная острота зрения (МКОЗ)): правый глаз =1,0, левый глаз =1,0. Тогда же выявлены БГ, манифестный тиреотоксикоз, претибиальная микседема. Не курит; наследственность отягощена по заболеваниям ЩЖ. Исходно антитела к рецепторам тиреотропного гормона (АТ-рТТГ) >40,00 МЕ/л. Объем ЩЖ — 18,41 мл. Консервативная терапия тиамазолом по схеме «блокируй», с мая 2023 г. в эутиреозе (ТТГ — 0,45 мМЕ/л). Сопутствующие заболевания: хронический тонзиллит вне обострения, хроническая железодефицитная анемия легкой степени, дислипидемия, фиброаденома молочной железы. В связи с сохраняющимися глазными симптомами в марте 2023 г. амбулаторно назначен преднизолон per os с 35 мг в сутки по убывающей схеме. В апреле 2023 г. пациентка консультирована офтальмологом «Клинической офтальмологической больницы им. В.П. Выходцева». МКОЗ: правый глаз=0,9, левый глаз=0,8. Экзофтальмометрия: OD=26 мм, OS=26 мм, р=122 мм. Ширина глазной щели (ШГЩ) OU 13 мм. Аппланационная тонометрия OD Po=22, ВГД OS Po=22. ЗН (МСКТ): OD/OS=4,5 мм.> Диагноз: «ЭОП тяжелой степени, активная (правый глаз CAS=5, левый глаз CAS=7). Оптическая нейропатия. Вторичное рестриктивное косоглазие. Бинокулярное двоение» (рис. 1).

**Figure:**
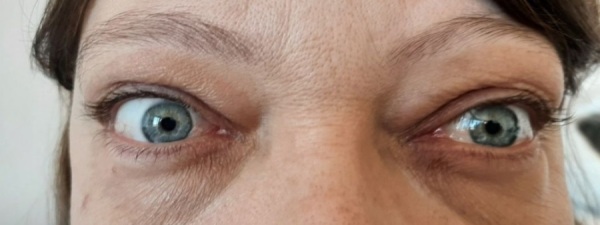
Рисунок 1. Внешний вид пациентки до начала пульс-терапии метилпреднизолоном.

Начат цикл пульс-терапии метилпреднизолоном (МП): 500 мг 2 раза в неделю — 2 недели, 750 мг в неделю — 4 недели. Исходно и далее неоднократно проведены телемедицинские консультации в ГНЦ РФ ФГБУ «НМИЦ эндокринологии» Минздрава России. На 7-й неделе после снижения дозы МП до 500 мг в неделю по достижении суммарной дозы МП 5250 мг пациентка направлена на контрольное обследование к офтальмологу. В связи со снижением остроты зрения на фоне снижения дозы МП интенсифицирована схема пульс-терапии до 500 мг МП 3 раза в неделю в течение двух недель. По улучшении доза МП снижена до 250 мг в неделю. Однако при офтальмологическом осмотре на 12-й неделе цикла отмечено резкое снижение зрения обоих глаз до 0,5. При контрольном обследовании через неделю (суммарная доза МП 8500 мг) — дальнейшее ухудшение зрения правого глаза до 0,3, левого глаза до 0,2, ВГД OD Po=35,0 ВГД OS Po=24,0. Экзофтальмометрия: правый глаз — 23 мм, левый глаз — 25 мм. Активность ЭОП обоих глаз по шкале CAS=5. Компьютерная периметрия (КП) OD: относительная центральная скотома, единичные относительные парацентральные скотомы; незначительное изменение показателя MD (Mean Deviation): -2,86dB; показатель PSD (Pattern Standard Deviation) в пределах нормы :1,63dB (рис. 2).

**Figure:**
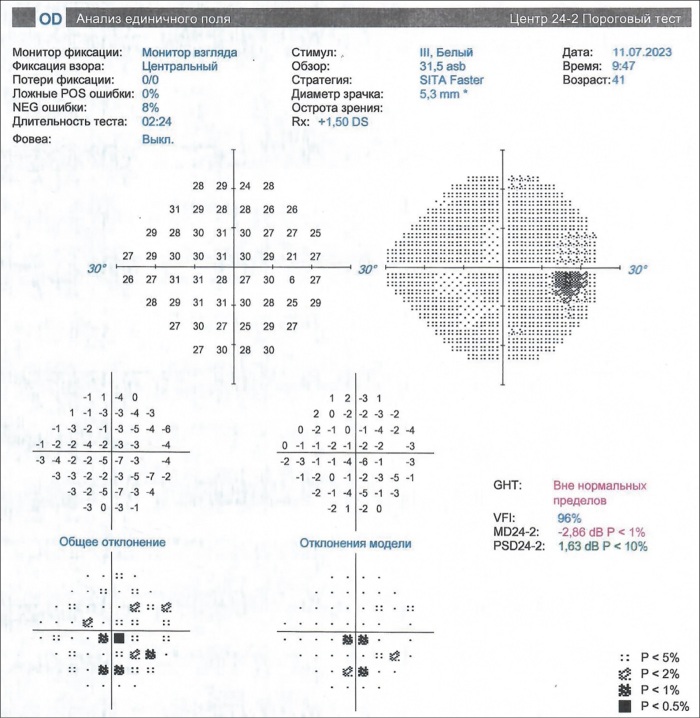
Рисунок 2. Компьютерная периметрия OD до начала терапии ТЦЗ; ZEISS Humphrey Field Analyzer.

КП OS: скотомы относительные и абсолютные центральная и парацентральные; умеренное изменение показателя MD (Mean Deviation): -4,23dB; показателя PSD (Pattern Standard Deviation): 2,24 dB (рис. 3).

**Figure:**
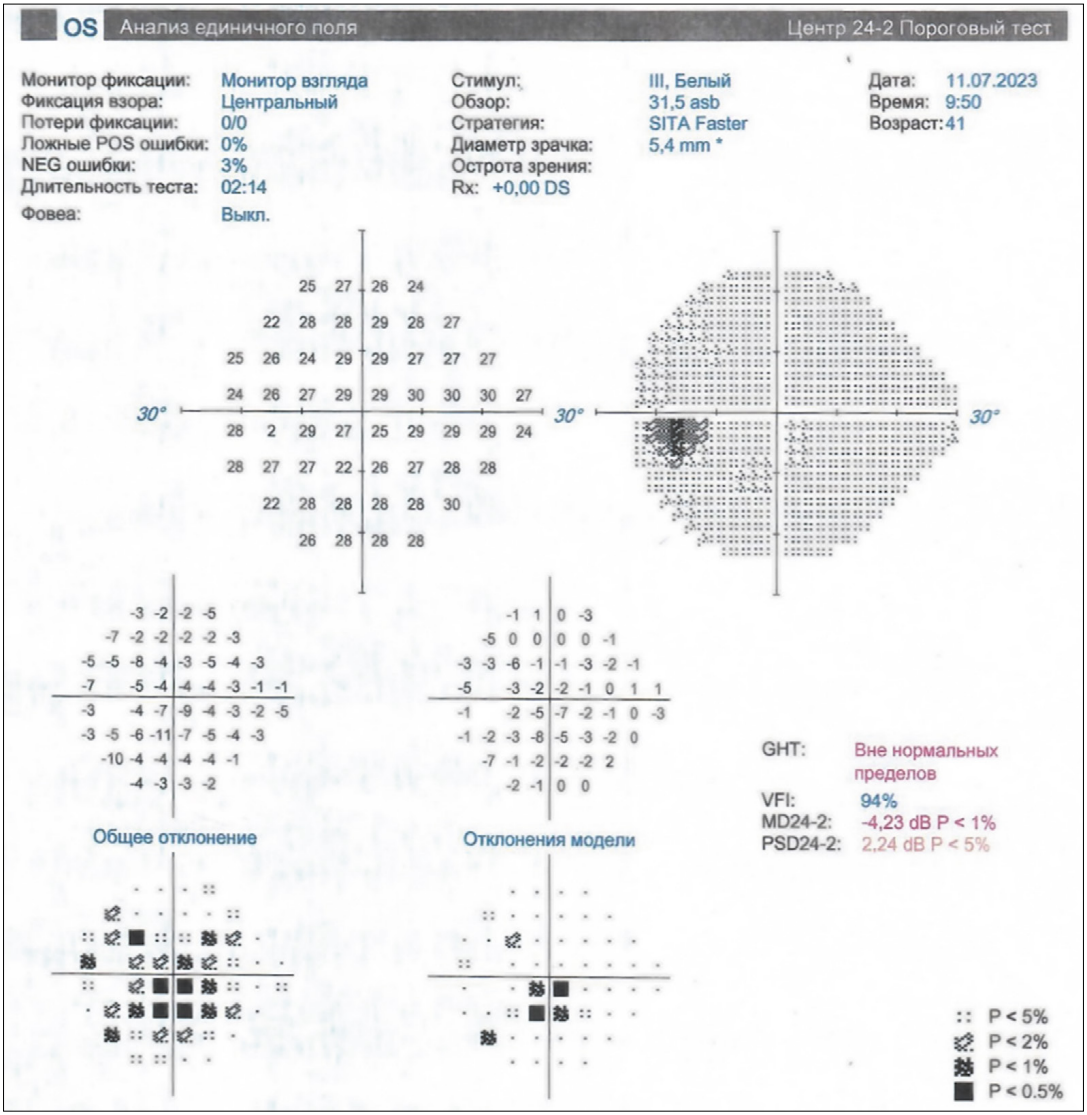
Рисунок 3. Компьютерная периметрия OS до начала терапии ТЦЗ; ZEISS Humphrey Field Analyzer.

МРТ орбит до начала терапии ТЦЗ: стрелками указаны увеличенные внутренние и наружные прямые мышцы: справа — 8,5 мм, 8,1 мм; слева — 7,5 мм, 7,5 мм соответственно (рис. 4).

**Figure:**
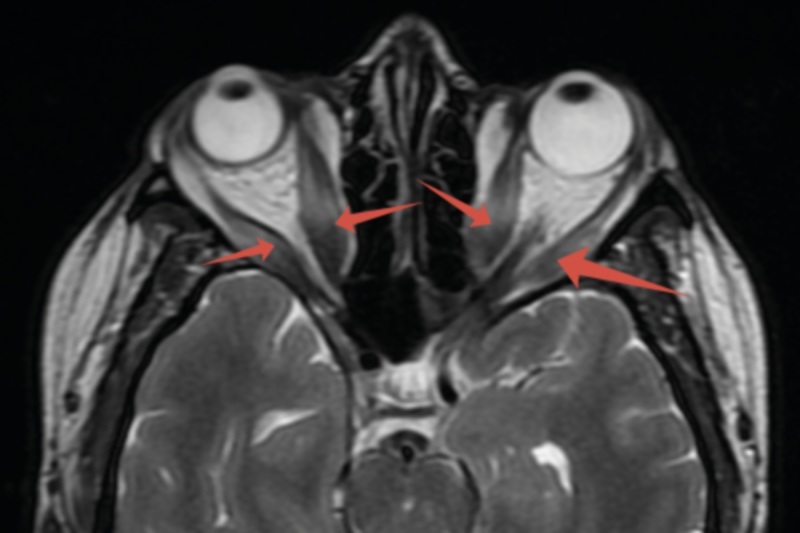
Рисунок 4. МРТ орбит до начала терапии ТЦЗ, доза МП 8500 мг. Стрелками указаны увеличенные внутренние и наружные прямые мышцы. Ax T2 FRFSE. Обращает внимание увеличение размеров прямых мышц (стрелки) и характерная для тяжелой ЭОП деформация медиальных стенок орбиты.

В связи с угрозой потери зрения, высокой дозой МП (8500 мг), резистентностью к ГК получены рекомендации ГНЦ РФ ФГБУ «НМИЦ эндокринологии» Минздрава России по введению ТЦЗ в соответствии с «The 2021 EUGOGO clinical practice guidelines for the medical management of Graves’ orbitopathy» и «Management of Thyroid Eye Disease: A Consensus Statement by the ATA and the ETA» в дозе 8 мг/кг внутривенно. 1-я доза ТЦЗ введена на 13-й неделе от начала введения МП. Суммарная доза МП составила 10 000 мг. Через 10 дней от введения 1-й дозы пациентка госпитализирована в ЭНЦ. При контрольном осмотре офтальмологом МКОЗ правого глаза — 0,9, левого глаза — 0,9. АТ-рТТГ — 10,0 МЕ/л, ТТГ — 0,34 мМЕ/л, АлАТ — 15,3 Е/л; АсАТ — 15,5 Е/л; ГГТ — 14 Е/л, общий объем ЩЖ — 22,4 мл. 2-я доза ТЦЗ введена с интервалом 4 недели. При контроле через неделю МКОЗ правого глаза — 0,5, левого глаза — 0,3, однако с некоторой положительной динамикой компьютерной периметрии. В начале сентября на правом глазу развился кератит вне оптической зоны, разрешившийся в течение 2-х недель. По разрешении кератита значительно улучшились показатели OS vis до 0,9, однако снизилась показатели OD vis до 0,3. Ввиду снижения остроты зрения на правом глазу до 0,3 пациентка направлена на декомпрессию орбиты. 19.09.2023 г. выполнена глубокая костная декомпрессия латеральной стенки OD в ФГБНУ «НИИГБ им. М.М. Краснова».> Через месяц МКОЗ правого глаза — 0,9, левого глаза — 1,0. Экзофтальм OD=19 мм, OS=22 мм, р=116 мм. ШГЩ OD=10 мм, OS=11 мм. Тонометрия по Маклакову ВГД OD Po=23,7 мм рт.ст, ВГД OS Po=23,0 мм рт.ст. ЗН (МРТ): OD=4,2 мм, OS=4,3 мм.> Активность ЭОП по шкале> CAS правый глаз=3, левый глаз=2. Поскольку сохранялись признаки активности ЭОП, подтвержденные данными МРТ орбит, введение ТЦЗ продолжено через 1,5 месяца после декомпрессии (через 2,5 мес от введения 2-й дозы). МРТ орбит после глубокой костной декомпрессии латеральной стенки OD, размеры внутренних и наружных прямых мышц: справа — 9,2 мм; 6,0 мм, слева — 8,8 мм; 5,5 мм соответственно (рис. 5).

**Figure:**
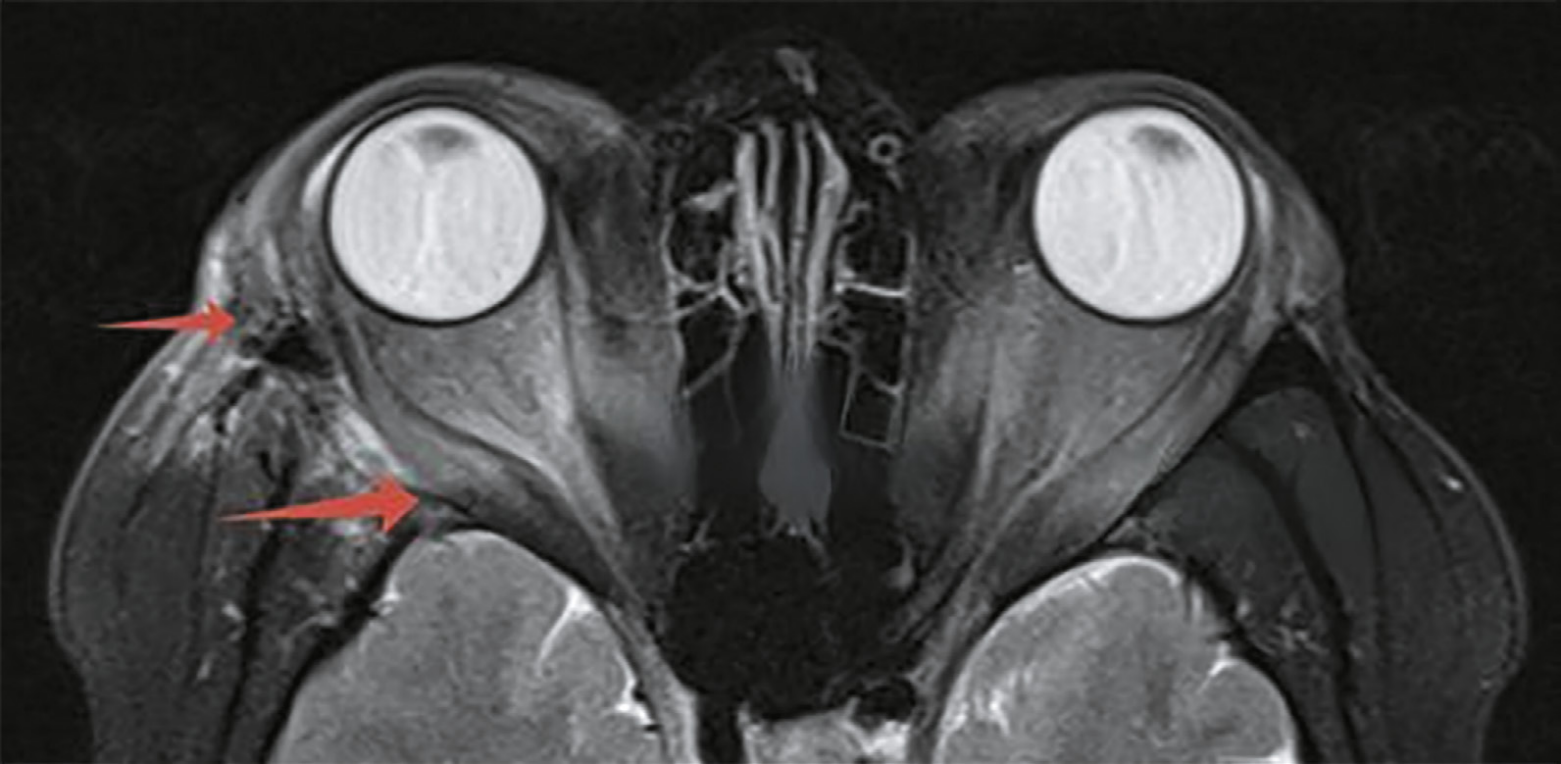
Рисунок 5. МРТ орбит после двух доз ТЦЗ и латеральной костной декомпрессии правой орбиты, доза МП 10000 мг. Ax T2 FRFSE. Стрелками показаны границы послеоперационного костного окна в наружной стенке справа, визуализируется выход наружной прямой мышцы в сформированное пространство.

С интервалом 4 недели введены 3-я и 4-я дозы ТЦЗ. Нежелательных реакций не зарегистрировано. Показатели печеночных трансаминаз и гамма-глутамилтрансферазы (ГГТ) при проведении системной терапии находились в референсном диапазоне: АлАТ — 9–15,3 Е/л; АсАТ — 14–15,5 Е/л; ГГТ — 13–14 Е/л. Весь период активного лечения составил около 8 месяцев. По завершении пульс-терапии в период лечения ТЦЗ мы также дважды применяли преднизолон per os до 15–20 мг/сутки, сроком до трех недель.

При контроле через 6 мес от начала терапии ТЦЗ МКОЗ правого глаза — 0,9 н/к, левого глаза — 1,0. Экзофтальмометрия p=116, OD=17 мм, OS=21 мм. ШГЩ OD=9 мм, OS=10 мм. Активность ЭОП обоих глаз по шкале CAS=1.

Через 9 мес МКОЗ правого глаза — 0,8, левого глаза — 1,0, ВГД OD Po=23,0 мм рт.ст., ВГД OS Po=23,0 мм рт.ст. Экзофтальмометрия p=116, OD=17 мм, OS=20 мм. ШГЩ OD=9 мм, OS=10 мм. Активность ЭОП обоих глаз по шкале CAS=1. АТ-рТТГ — 1,8 МЕ/л, ТТГ — 2,8 мМЕ/л. Рекомендованы гипотензивные капли постоянно.

Препарат ТЦЗ относится к категории «off label», перед каждой инфузией получены рекомендации ГНЦ РФ ФГБУ «НМИЦ эндокринологии» Минздрава России, проведены консилиум и врачебная комиссия, оформлено информированное согласие пациентки. При данной нозологии на территории РФ препарат применен впервые.

## Исход и результаты последующего наблюдения

Мы продолжаем динамическое наблюдение пациентки. При офтальмологическом контроле через 15 мес от начала терапии ТЦЗ МКОЗ правого глаза — 0,8, левого глаза — 1,0. Активность ЭОП обоих глаз по шкале CAS=0. Экзофтальмометрия p=116, OD=18 мм, OS=21 мм. ШГЩ OD=7 мм, OS=10 мм (рис. 6).

**Figure:**
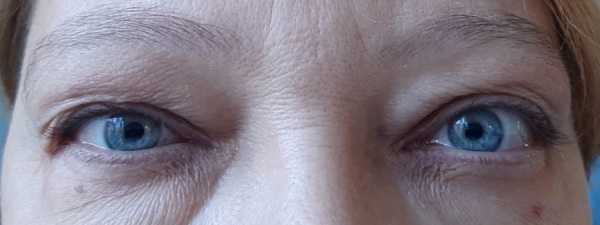
Рисунок 6. Внешний вид пациентки через 15 мес от начала терапии ТЦЗ.

Сохраняется легкая диплопия при взгляде вдаль. Медикаментозный эутиреоз на фоне терапии тиамазолом. Пациентка вернулась к обычной жизни по завершении иммуносупрессивной терапии, трудоспособна. Планируется проведение радиойодтерапии болезни Грейвса.

## ОБСУЖДЕНИЕ

Резистентность к ГК — особый аспект ведения пациентов с ЭОП. В настоящее время не разработаны четкие общепризнанные критерии резистентности к ГК при этом заболевании. Имеющиеся в литературе данные определяют резистентность посредством таких опорных точек как изменение CAS ≤ 2 при введении трех последовательных доз МП 500 мг и рецидив по завершении курса пульс-терапии [16]. Однако известные ограничения шкалы CAS [11][12][21] не позволяют адекватно оценить ответ на ГК у некоторых категорий пациентов. При ведении пациентов с ОН оценка эффективности противовоспалительной терапии должна включать прежде всего показатели зрительных функций. Отсутствие динамики или снижение остроты зрения у этих пациентов является одним из критериев резистентности к ГК.

В рекомендациях EUGOGO акцентирован контроль ответа на терапию первой линии через 6 недель от начала пульс-терапии ГК [11]. У нашей пациентки отмечались отчетливые признаки резистентности к ГК уже на 7-й неделе пульс-терапии. Однако известные риски и сложности в применении препаратов “off label” привели к вынужденно отсроченному началу второй линии терапии.

В нашем случае невозможно четко разграничить эффекты ГК и ТЦЗ, поскольку максимальный период полезного действия МП составляет до 6–12 недель [11][12][18]. При наличии угрозы потери зрения клинической целью была не оценка изолированного ответа на ТЦЗ, а максимально быстрое терапевтическое воздействие. Однако резко отрицательная динамика по достижении высокой суммарной дозы МП, вынужденно превышающей рекомендованную безопасную дозу, предположительно минимизирует вклад ГК в полученные результаты.

Влияние тиреоидного статуса и антитиреоидной терапии (тиамазол) в данном случае вероятно также было минимальным, так как весь указанный период пациентка находилась в эутиреозе.

Имеющиеся в литературе критерии абсолютного ответа на терапию ТЦЗ — достижение CAS≤1 и АТ-рТТГ≤10 МЕ/л [18].

Улучшение остроты зрения. На фоне лечения у нашей пациентки достигнуто полное восстановление vis OS до 1,0 с повышением на 0,7 от начала терапии ТЦЗ. Значительные риски необратимого нарушения зрительных функций вследствие длительной компрессии ЗН OD мы сочли поводом для приостановления консервативной терапии в пользу неотложной глубокой декомпрессии наружной стенки орбиты. Возможно, более раннее начало терапии второй линии позволило бы избежать хирургического вмешательства. Продолжение консервативной терапии после костной декомпрессии справа способствовало стабилизации результатов оперативного лечения OD без ухудшения. Результаты ранее представленного проспективного когортного исследования продемонстрировали высокую эффективность ТЦЗ при компрессии ЗН без применения декомпрессии орбит с улучшением остроты зрения в среднем на 0,4 [21]. В других исследованиях с ТЦЗ также участвовали пациенты с ОН [15–20]. ТЦЗ применялся в случаях рефрактерной к ГК ЭОП, в сочетании с лучевой терапией орбит или без нее, после применения ГК и декомпрессии и даже в качестве терапии первой линии, когда другие варианты не были рекомендованы.

Снижение CAS. Мы получили значительное снижение CAS: через 3 мес от начала терапии ТЦЗ на 2–3 балла, через 6–9 мес. на 4 балла, через 15 мес на 5 баллов. По результатам представленных отчетов, в исследованиях со статистическим анализом после лечения ТЦЗ наблюдалось снижение показателя CAS по крайней мере на 3 балла [15–22].

Уменьшение экзофтальма. В ходе лечения нами отмечено значимое уменьшение экзофтальма: через 3 мес. от начала терапии ТЦЗ на 3 мм, через 6 мес на 4 мм, через 15 мес на 5 мм в отношении неоперированного глаза. По имеющимся данным, в исследованиях наблюдалось снижение степени экзофтальма не менее чем на 3 мм [15–22]. Недавний метаанализ предполагает, что ТЦЗ может быть наиболее эффективен в отношении экзофтальма, превосходя по эффекту тепротумумаб и ритуксимаб [22]. Однако результаты единственного рандомизированного контролируемого исследования не показали статистически значимого уменьшения экзофтальма [18].

Уменьшение диплопии. Мы не оценивали диплопию как критерий ответа на ТЦЗ, однако через 1,5 мес. от начала терапии пациентка отметила отсутствие диплопии при взгляде вблизи, имевшуюся ранее. По имеющимся данным, эффективность ТЦЗ в отношении диплопии весьма неоднозначна. Результаты метаанализа демонстрируют, что ТЦЗ не является оптимальным средством для устранения диплопии [22].

Снижение АТ-рТТГ. Мы не определяли АТ-рТТГ с достаточной для оценки динамики частотой по экономическим причинам, однако через 9 мес от начала терапии ТЦЗ этот показатель снизился до 1,8 МЕ/л от 10,0 МЕ/л.

## Нежелательные реакции

Исходно до начала цикла пульс-терапии и весь период лечения проводился стандартный рекомендованный общеклинический лабораторно-инструментальный контроль. По достижении суммарной дозы МП 8000 мг было проведено дополнительное обследование с целью тщательного мониторинга нежелательных реакций (ЭхоКГ, УЗИ абдоминальное, расширенная коагулограмма).

В период терапии ТЦЗ накануне введения препарата и ежедневно после в течение недели проводился контроль общего и биохимического анализов крови, общего анализа мочи, ЭКГ. Прокальцитонин определяли исходно и через неделю от введения препарата. Также после принятия решения о продолжении терапии ТЦЗ в плановом порядке по результатам введения первой дозы пациентка была детально обследована в соответствии с инструкцией к препарату. Проведено обследование с целью исключения латентной туберкулезной инфекции, получено разрешение профильной врачебной комиссии. Также проведена ТАБ образования молочной железы, получено разрешение онколога на проведение терапии ТЦЗ. Перед введением первой дозы указанные обследования не выполнены ввиду неотложной ситуации, угрозы потери зрения. Через год от начала терапии по результатам УЗИ молочных желез образований не выявлено.

После введения первой дозы ТЦЗ на следующий день зафиксировано снижение гемоглобина на 22 г/л от исходного с соответствующим снижением количества эритроцитов. При дальнейшем контроле показатели вернулись к исходным в течение нескольких дней. Лабораторных признаков внутрисосудистого гемолиза не зафиксировано. Также исключены возможные источники кровотечения, в т.ч. при ЭФГДС. Стоит учесть, что в день введения первой дозы ТЦЗ и на следующий также введено 2 дозы МП по 500 мг, с соответствующим объемом инфузий. Введение 2, 3, 4 доз ТЦЗ проводилось без сопровождения МП, и далее подобных реакций со стороны эритроцитарного звена не отмечалось. Учитывая изложенное, мы не расценили данный эпизод как нежелательную реакцию, связанную с ТЦЗ.

Гиперхолестеринемия, как один из вероятных побочных эффектов ТЦЗ, имелась у пациентки до начала введения и при контроле динамики в липидном спектре мы не выявили. Статины не назначались в связи с низким риском по шкале SCORE2.

Стоит учесть, что в представленных ранее отчетах альтернативное лечение ТЦЗ оказалось неэффективным у 8 пациентов (8 орбит), резистентных к внутривенному введению МП. Группа из семи таких пациентов (7 орбит) была проанализирована в рамках одного ретроспективного исследования, которое не выявило значительного улучшения остроты зрения после 1 года лечения ТЦЗ [8][14]. Кроме того, отсутствие лабораторных предикторов ответа на терапию ТЦЗ весьма осложняет более точный отбор среди пациентов, нуждающихся в терапии 2-й линии [5][6][8][9][18][21][22].

## ЗАКЛЮЧЕНИЕ

Нами представлен впервые в РФ клинический случай применения тоцилизумаба при тяжелой ЭОП с оптической нейропатией, рефрактерной к глюкокортикоидам. Несмотря на эффективность препарата, потребовалось выполнение глубокой латеральной костной декомпрессии орбиты в кратчайшие сроки после верификации отрицательной динамики оптической нейропатии. Представленный клинический случай наглядно иллюстрирует практическую потребность в альтернативных препаратах для пациентов с ЭОП. Тоцилизумаб может быть эффективным во второй линии терапии при тяжелой ЭОП с ОН, резистентной к ГК.

## ДОПОЛНИТЕЛЬНАЯ ИНФОРМАЦИЯ

Источники финансирования. Работа выполнена по инициативе авторов без привлечения финансирования.

Конфликт интересов. Авторы декларируют отсутствие явных и потенциальных конфликтов интересов, связанных с содержанием настоящей статьи.

Участие авторов. Все авторы одобрили финальную версию статьи перед публикацией, выразили согласие нести ответственность за все аспекты работы, подразумевающую надлежащее изучение и решение вопросов, связанных с точностью или добросовестностью любой части работы.

Согласие пациента. Пациент добровольно подписал информированное согласие на публикацию персональной медицинской информации в обезличенной форме в журнале «Проблемы эндокринологии».

Благодарности. Авторы выражают признательность В.В. Клишину, Д.В. Дячек и Д.Р. Мингулову за консультации и техническую помощь.

## References

[cit1] Antonelli Alessandro, Fallahi Poupak, Elia Giusy, Ragusa Francesca, Paparo Sabrina Rosaria, Ruffilli Ilaria, Patrizio Armando, Gonnella Debora, Giusti Claudia, Virili Camilla, Centanni Marco, Shoenfeld Yehuda, Ferrari Silvia Martina (2020). Graves' disease: Clinical manifestations, immune pathogenesis (cytokines and chemokines) and therapy. Best Practice & Research Clinical Endocrinology & Metabolism.

[cit2] Sviridenko N.Yu. Glava 4. Endokrinnaya oftal'mopatiya - autoimmunnaya patologiya glaz / N. Yu. Sviridenko // Autoimmunnaya patologiya shchitovidnoi zhelezy i endokrinnaya oftal'mopatiya. — Moskva : Obshchestvo s ogranichennoi otvetstvennost'yu «Tipografiya «Pechatnykh Del Master», 2020. – S. 68-73.

[cit3] Yoon Jin Sook, Kikkawa Don O. (2022). Thyroid eye disease. Taiwan Journal of Ophthalmology.

[cit4] Men Clara J., Kossler Andrea L., Wester Sara T. (2021). Updates on the understanding and management of thyroid eye disease. Therapeutic Advances in Ophthalmology.

[cit5] Hodgson Nickisa M., Rajaii Fatemeh (2019). Current Understanding of the Progression and Management of Thyroid Associated Orbitopathy: A Systematic Review. Ophthalmology and Therapy.

[cit6] Moi Laura, Hamedani Mehrad, Ribi Camillo (2021). Long‐term outcomes in corticosteroid‐refractory Graves' orbitopathy treated with tocilizumab. Clinical Endocrinology.

[cit7] Brovkina A.F. Opticheskaya neiropatiya i otechnyi ekzoftal'm: simptom ili oslozhnenie? // Oftal'mol. vedomosti. 2020. №1. URL: https://cyberleninka.ru/article/n/opticheskaya-neyropatiya-i-otyochnyy-ekzoftalm-simptom-ili-oslozhnenie

[cit8] Pelewicz-Sowa M., Miśkiewicz P. (2023). Dysthyroid optic neuropathy: emerging treatment strategies. Journal of Endocrinological Investigation.

[cit9] Dolman P. J. (2020). Dysthyroid optic neuropathy: evaluation and management. Journal of Endocrinological Investigation.

[cit10] Vorontsov A.V., Sviridenko N.Yu., Remizov O.V. Glava 9. Vizualiziruyushchie metody issledovaniya orbit v diagnostike endokrinnoi oftal'mopatii. – 2020.

[cit11] Bartalena L, Kahaly G J, Baldeschi L, Dayan C M, Eckstein A, Marcocci C, Marinò M, Vaidya B, Wiersinga W M, _ _, _ _, Ayvaz Goksun, Konuk Onur, Ciric Jasmina, Beleslin Bijliana, Boschi Antonella, Cristina Burlacu Maria, Morris Dan, Le Moli Rosario, Marino Antonio, McKee Justin, Zammit Nicola, Führer Dagmar, Pereni Ioana, Schittkowski Michael, Raddatz Dirk, Lee Vickie, Meeran Karim, Abeillon Juliette, Soui Thcong Thia, Ponto Katharina, Muller Ilaria, Currò Nicola, Hintschich Christoph, Gärtner Roland, Pearce Simon, Clarke Lucy, Brix Thomas, Bechtold Dorte, Rudovfsky Gottfried, Fichter Nicole, Du Pasquier Laurence, Meney Julia, Menconi Francesca, Lanzolla Giulia, Sundar Gangadhara, Peiling Yung Samantha, Boboridis Kostas, Anagnostis Panagiotis, Pérez Lopez Marta, Javier Sanchez Carlos, Laura Tanda Maria, Donati Simone, Papp Andrea, Li Shuren, Jablonska Anna, Miskiewicz Piotr, Juri Mandic Jelena, Baretic Maja (2021). The 2021 European Group on Graves’ orbitopathy (EUGOGO) clinical practice guidelines for the medical management of Graves’ orbitopathy. European Journal of Endocrinology.

[cit12] Burch Henry B., Perros Petros, Bednarczuk Tomasz, Cooper David S., Dolman Peter J., Leung Angela M., Mombaerts Ilse, Salvi Mario, Stan Marius N. (2022). Management of Thyroid Eye Disease: A Consensus Statement by the American Thyroid Association and the European Thyroid Association. Thyroid.

[cit13] Grusha Y.O., Kochetkov P.A., Sviridenko N.Yu., Kolodina A.S., Dzamikhov I.K. (2024). Bony orbital decompression in thyroid eye disease. Russian Annals of Ophthalmology.

[cit14] Bessmertnaya E. G., Mikheenkov A. A., Kolodina A. S., Aksenova T. N., Babaeva D. M., Grusha Ya. O., Sviridenko N. Yu. (2024). Phasing and continuity of the treatment of thyroid eye disease in patients with Graves’ disease. Problems of Endocrinology.

[cit15] Moi Laura, Hamedani Mehrad, Ribi Camillo (2021). Long‐term outcomes in corticosteroid‐refractory Graves' orbitopathy treated with tocilizumab. Clinical Endocrinology.

[cit16] Sánchez-Bilbao Lara, Martínez-López David, Revenga Marcelino, López-Vázquez Ángel, Valls-Pascual Elia, Atienza-Mateo Belén, Valls-Espinosa Beatriz, Maiz-Alonso Olga, Blanco Ana, Torre-Salaberri Ignacio, Rodríguez-Méndez Verónica, García-Aparicio Ángel, Veroz-González Raúl, Jovaní Vega, Peiteado Diana, Sánchez-Orgaz Margarita, Tomero Eva, Toyos-Sáenz de Miera Francisco J., Pinillos Valvanera, Aurrecoechea Elena, Mora Ángel, Conesa Arantxa, Fernández-Prada Manuel, Troyano Juan A., Calvo-Río Vanesa, Demetrio-Pablo Rosalía, González-Mazón Íñigo, Hernández José L., Castañeda Santos, González-Gay Miguel Á., Blanco Ricardo (2020). Anti-IL-6 Receptor Tocilizumab in Refractory Graves’ Orbitopathy: National Multicenter Observational Study of 48 Patients. Journal of Clinical Medicine.

[cit17] Mehmet Aysel, Panagiotopoulou Eirini Kanella, Konstantinidis Aristeidis, Papagoras Charalampos, Skendros Panagiotis, Dardabounis Doukas, Mikropoulou Athanasia Maria, Labiris Georgios (2021). Α Case of Severe Thyroid Eye Disease Treated with Tocilizumab. Acta Medica (Hradec Kralove, Czech Republic).

[cit18] Pérez-Moreiras José V., Varela-Agra María, Prada-Sánchez M. Consuelo, Prada-Ramallal Guillermo (2021). Steroid-Resistant Graves’ Orbitopathy Treated with Tocilizumab in Real-World Clinical Practice: A 9-Year Single-Center Experience. Journal of Clinical Medicine.

[cit19] Pascual-Camps I., Molina-Pallete R., Bort-Martí M. A., Todolí J., España-Gregori E. (2018). Tocilizumab as first treatment option in optic neuropathy secondary to Graves’ orbitopathy. Orbit.

[cit20] Maldiney Thomas, Deschasse Clémence, Bielefeld Philip (2018). Tocilizumab for the Management of Corticosteroid-Resistant Mild to Severe Graves’ Ophthalmopathy, a Report of Three Cases. Ocular Immunology and Inflammation.

[cit21] Habroosh Fatima A., Albrashdi Safiya S., Alsaadi Ahmed H., Eatamadi Habibullah (2024). Tocilizumab use for optic nerve compression in thyroid eye disease: a prospective longitudinal cohort. International Ophthalmology.

[cit22] Duarte Ana F., Xavier Naiara F., Sales Sanz Marco, Cruz Antonio A. V. (2024). Efficiency and Safety of Tocilizumab for the Treatment of Thyroid Eye Disease: A Systematic Review. Ophthalmic Plastic & Reconstructive Surgery.

